# Calcified extra-axial cavernous malformation arising from lower cranial nerves: A case report and literature review

**DOI:** 10.1097/MD.0000000000024566

**Published:** 2021-02-05

**Authors:** Daibo Ke, Xueyun Deng, Xiang Li, Jiuhong Li, Xuhui Hui

**Affiliations:** a Department of Neurosurgery, West China Hospital of Sichuan University, Chengdu, Sichuan; b Department of Neurosurgery, Nanchong Central Hospital, The Second Clinical Medical College of North Sichuan Medical College, Nanchong, China.

**Keywords:** calcification, cavernous malformations, cranial nerves

## Abstract

**Rationale:**

Extra-axial cavernous malformations (ECMs) arising from cranial nerves (CNs) are rare. Complete “en bloc” lesion resection and hemosiderin-stained tissue preservation remain the standard treatment, while a different strategy may be needed when the lesion is highly calcified . We report the 3rd calcified ECM-CN and review the clinical features and surgical strategy for this rare condition considering previous literature.

**Patient concerns:**

We present a 52-year-old woman with a calcified lesion located in the right lower 1/3 of the cerebellopontine angle.

**Diagnosis:**

The diagnosis was calcified ECM-CNs according to the pathological and radiological features.

**Interventions:**

A posterior midline craniotomy was performed, and piecemeal resection of the lesion was carried out. Subtotal resection of the lesion was achieved with a small piece left in situ.

**Outcomes:**

No symptom or lesion-related recurrence was found during 28 months of follow-up.

**Lessons:**

Calcified ECM-CNs are unique cavernous malformations arising from CNs. Piecemeal resection and subtotal or near-total excision are 2 major aspects that differ from the surgical strategy for general ECM-CNs.

## 1 Introduction

Cavernous malformations (CMs) constitute 10% to 20% of all vascular malformations in the central nervous system. They most commonly occur in the subcortex, basal ganglia, cerebellar hemisphere and brain stem.^[1,2]^ Extra-axial CMs arising from cranial nerves (CNs) (ECM-CNs) are rare, with fewer than 80 cases reported previously.^[3,4]^ While complete “en bloc” resection of the lesion with preservation of the hemosiderin-stained tissue is highly recommended for most EAC-CNs, a different surgical procedure exists for ECM-CNs with calcification, which is extremely rare, with only 2 cases reported previously.^[5,6]^ Here, we report the third calcified ECM-CN and review the clinical features and surgical strategy for this rare condition considering previous literature (Table 1).

**Table 1:** Clinical data of calcified ECM-CNs.

Article	Age/Gender	Clinical Presentation	Imaging	Surgery	Outcome
Albanese et al 2009^[5]^	48/M	Gait instability and loss in tone of voice for 5 months	CT: An extremely calcified mass in the right CPAMRI: Hypointense on T1 and T2 with heterogeneous enhancement	Right retrosigmoid approach;An extremely calcified mass was tightly adherent to the lower CNs;Minimal remnants of calcifi-cations are left on the surface of lower CNs with STR of the lesion.	Significant improvement in the tone of voice and no surgery related complication was found.
Nair et al 2014^[6]^	59/M	Hoarseness, swallowing disorder, nasal regurgitation for 5 months	CT: Hyperdense lesion in the right CPAMRI: Hyperintense on T1 and hypointense on T2.	Right retromastoid approach;A calcified lesion with inferior compression of lower CNs GTR was achieved.	Lower cranial nerve function remained the same.
Present case	52/F	Progressive dysphagia, choking and left lower limb weakness for 6 months	CT: Mulberry-shaped hyperdense lesion in the right PFAMRI: Hypointense on T1 and T2 with homogenous enhancement	Posterior midline approach;The lesion was densely adhered to the proximal part of lower CNs;A small piece of residual was left in situ with STR of the lesion	Improved significantly without recurrence 18 months after surgery.

## 2 Case report

**Figure 1:**
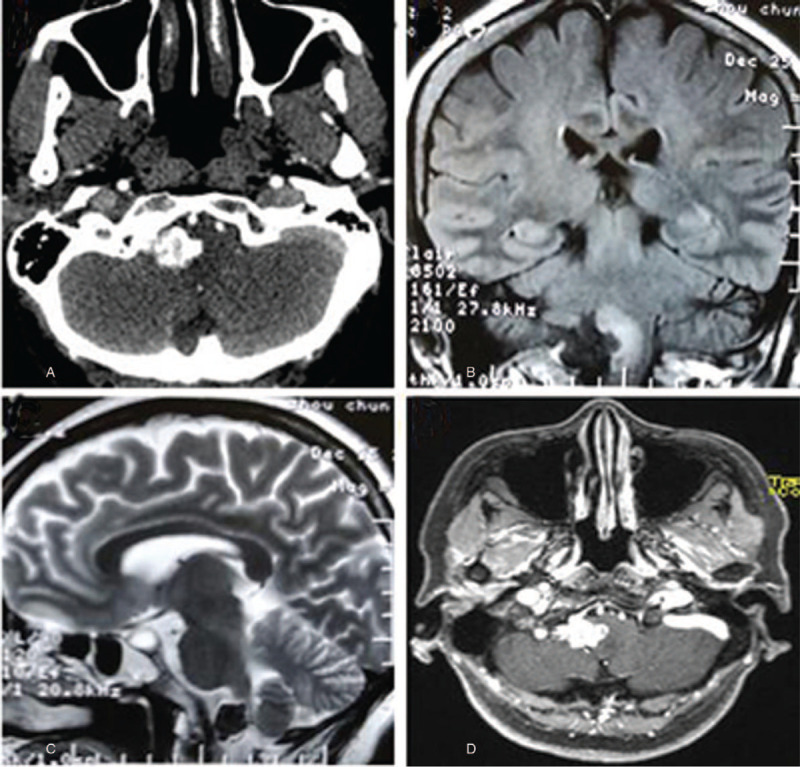
(A) Head computed tomography scan showing a lesion with a hyperattenuated signal in the right jugular foramen area. (B) Coronary T1 and (C) sagittal T2 MRI showing a hypointense lesion with edema of the adjacent brainstem parenchyma. (D) The lesion was homogenously enhanced after gadolinium administration.

**Figure 2:**
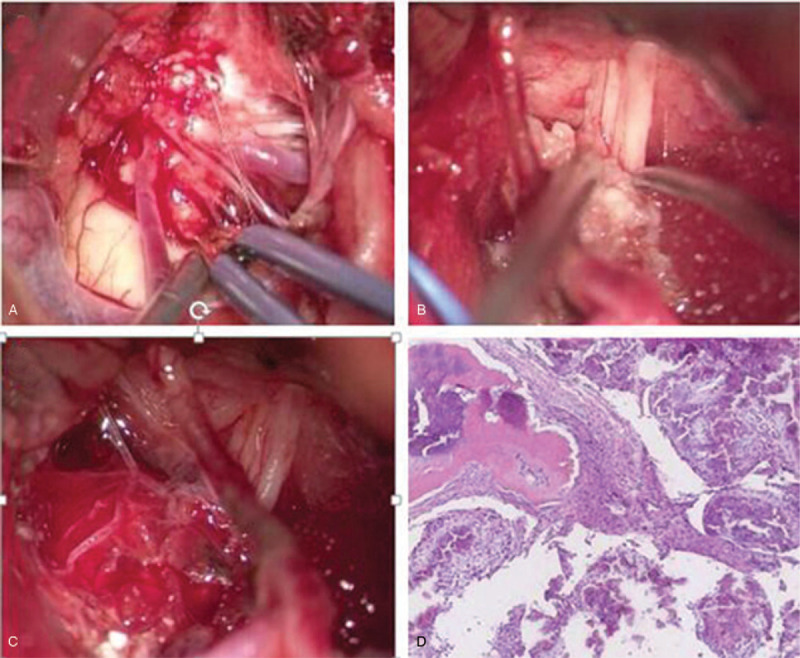
(A)The cavernous malformation was covered by rising branches of cervical nerves and posterior inferior cerebellar artery. (B) After exposing and partially resecting the lesion, it was found to be densely adhered to the lower CNs. (C) The lesion was subtotally resected with a small residual piece remaining in in the proximal part of the lower CNs. (D) Histopathological findings showing thin-walled vessels, intravascular calcification and thrombotic debris.

**Figure 3:**
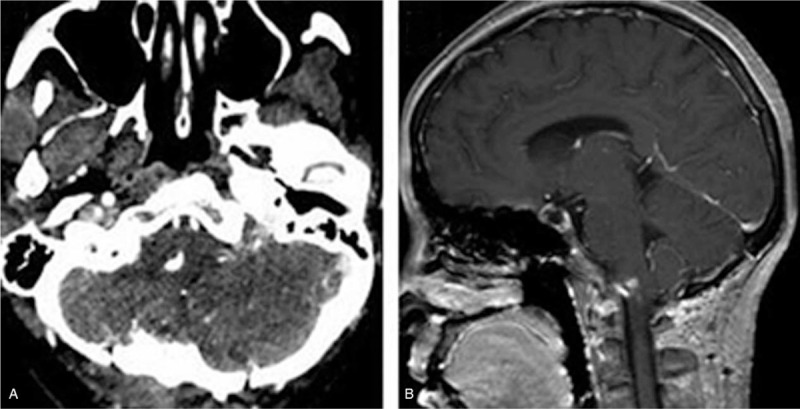
(A)Postoperative CT showing a subtotal resection of the lesion. (B) Follow-up brain MRI showing that residual lesion remained stable at 28 months after surgery.

A 52-year-old woman presented with a 6-month history of progressive dysphagia, choking and left lower limb weakness. Physical examination revealed that the tongue deviated to the right side with remarkable waste on the left side, and grade 2 paresis on the left lower extremity. Head computed tomography (CT) showed a mulberry-shaped hyperdense mass measuring 13x20x18 mm in the right lower 1/3 of the cerebellopontine angle (Fig. 1A). The lesion was not adherent to the dural surface, and no obvious thickening or invasion of the petrosal bone was found. On brain magnetic resonance imaging (MRI), the lesion showed hypointensity on T1- and T2-weighted images with obvious edema of the adjacent brainstem parenchyma, which was homogenously enhanced after gadolinium injection (Fig. 1 B-D) and arise or protruded into the brainstem following the lower CN or arachnoid space. A posterior midline craniotomy was performed. Intraoperatively, a totally calcified brownish lesion was observed. The lesion was covered by cervical nerve branches and densely adhered to the proximal portion of the lower CNs (Fig. 2 A-B); subtotal resection was achieved with a small piece of lesion left in situ (Fig. 2C). Microscopically, the lesion was composed of variable sized vessels which were lined by endothelial cells and separated by connective fibrous tissue. Irregular areas of dense calcification, thrombotic debris and blood were present in the vessels (Fig. 2D). No intervening neural tissue was found, and a diagnosis of calcified CM was made. The preoperative symptoms improved significantly 3 weeks after surgery. After 28 months of follow-up, the patient was free from symptom recurrence, and follow-up brain CT (Fig. 3A) and MRI (Fig. 3B) showed that the residual lesion remained stable.

## 3 Discussion

ECMs arising from CNs are rare and have been reported to mostly affect the facial/vestibulocochlear complex (CN VII and CN VIII),^[3,4,7]^ optic nerve (CN II) ^[8–10]^ and trochlear nerve (CN V).^[11–13]^ Its pathological features are mostly the same as those of CMs in other locations of the CNS, including mulberry-like conglomeration of thin walled vascular sinusoids lined by a single layer of endothelium, which may or may not be thrombosed.^[14,15]^ For calcified CMs, except for the same features of regular CMs, calcified spherical bodies were generally found laid down in the walls of the involved vessels. Vaquero J et al^[16]^ and Kobayashi H et al^[17]^ suggested that the mechanism of calcification might be calcium deposition in the walls of progressively closed small arteries and degenerated areas related to localized rebleeding from these abnormal vessels. Although uncertainty remains, this hypothesis helps to explain the predilection of calcified ECM-CNs to involve the lower CNs, which have a relatively poor blood supply compared with the optic pathway and VII-VIII complex.^[5,6]^

Typical MRI patterns of CM are a well-circumscribed lesion with a central reticulated core of heterogeneous signal intensity and a peripheral hypointense rim in both T1- and T2 weighted images, with slight enhancement after gadolinium injection.^[10,18,19]^ However, these representative MRI features, especially the so-called “black rim”, are usually absent in ECMs, which makes the radiological diagnosis of CM challenging. Therefore, most extra-axial cavernomas may be diagnosed as other common tumors such as meningioma and schwannoma preoperatively.^[3,11,20,21]^ In contrast to MRI, CT has an obvious advantage for calcification analysis and leads to a better evaluation of calcified lesions.^[5,6,17]^ In this report, the lesion was easily recognized as a calcified lesion by evident hyperdense signals on CT and hypointensity on both T1- and T2 weighted images, however, it cannot be definitely diagnosed as CM because neither a reticulated core nor a “black rim” were found.

Surgical excision is the standard of care for symptomatic cavernomas in a noneloquent location.^[14,22,23]^ Although cleavage between the cavernoma and CNs is obscured and the CNs are usually found to be enclosed or tightly adhered to the cavernoma, en bloc resection of the cavernoma is recommended for most ECM-CNs to avoid severe bleeding.^[3,8,10]^ However, strict en bloc resection is very difficult and seems unnecessary for calcified ECM-CNs, especially those that are tightly adhered to adjacent structures. In contrast, skillful microdissection approaches with piecemeal mass resection are a more favorable strategy.^[5,6]^ In the 3 reported cases of calcified ECM-CNs, all patients experienced evident improvement of their preoperative symptoms and functional defects.

Furthermore, compared with common cavernomas, calcified CMs are considered a more benign form associated with a lower risk of tumor growth and hemorrhage.^[16,24,25]^ According to previous studies, the hemorrhage rate is approximately 0.25% per patient-year for common cavernomas, and recurrence can be found in patients with incomplete tumor resection;^[26–28]^ however, no recurrence or hemorrhagic characteristics have been observed for calcified cavernomas. Therefore, the use of near-total or subtotal resection for these lesions is acceptable to avoid further injury to fragile CNs. In the three reviewed cases of calcified ECM-CN with different grades of calcification, minimal remnants tightly adhered to the nerves were left in situ in all three patients. All patients achieved stable or improved neurological function without evidence of recurrence.

## 4 Conclusions

Calcified ECM-CNs are unique cavernomas involving CNs. Piecemeal resection and subtotal or near-total excision are 2 major aspects that differ from the surgical strategy for general ECM-CNs.

## Author contributions

All authors have made substantial contributions to this case report and approved submission to this journal.

**Conceptualization:** Daibo Ke, Xueyun Deng, Xuhui Hui.

**Formal analysis:** Xiang Li, Jiuhong Li.

**Methodology:** Daibo Ke, Xueyun Deng, Jiuhong Li.

**Supervision:** Xuhui Hui.

**Writing – original draft:** Daibo Ke, Xueyun Deng, Xiang Li.

**Writing – review & editing:** Daibo Ke, Xuhui Hui.
